# Mapping what matters: Integrating PPGIS into social impact assessment for mine planning and closure

**DOI:** 10.1007/s13280-025-02252-3

**Published:** 2025-10-16

**Authors:** Kamila Svobodova, Jo-Anne Everingham

**Affiliations:** 1 Centre for Social Responsibility in Mining, Sustainable Minerals Institute, The University of Queensland, Saint Lucia, QLD 4072 Australia; 2 Faculty of Environmental Sciences, Czech University of Life Sciences Prague, 16500 Prague, Suchdol, Czech Republic

**Keywords:** Community values, GIS, Justice, Participatory planning, Resource development, Spatial science

## Abstract

Community values toward landscapes, particularly before mining begins, are often overlooked in conventional social impact assessments in the mining industry. These emotional, cultural and relational values tend to be intangible and difficult to incorporate into formal planning tools. We propose public participation geographic information systems (PPGIS) as an underutilized method to address this gap. Rooted in participatory planning and spatial analysis, PPGIS offers a unique way to integrate landscape values into social impact assessments. When applied early in the mine lifecycle, PPGIS can inform both project design and closure planning, ensuring that social and cultural dimensions of landscapes are considered from the outset.  It aligns with regulatory requirements for meaningful community engagement, baseline assessment and closure visioning, and can strengthen both procedural fairness and social licence to operate. By combining technical data with local knowledge, PPGIS can support more inclusive, place-based and value-driven approaches to mine development and closure.

## Introduction: Mapping what matters before it is gone

The global mining industry is undergoing significant change, with mines opening and closing in response to intensified demand for critical minerals essential to low-carbon and high technology transitions (Svobodova et al. 2022). In this dynamic context, social impact assessment (SIA) plays a critical role in identifying, monitoring and managing the social and cultural consequences of mining projects. Although SIA remains a key tool for predicting impacts and informing approvals, it also plays an essential role in managing social issues throughout the entire project lifecycle, from planning to post-closure (Vanclay et al. 2015). As a background study to assess values in the physical and visual environment and the potential impacts of landscape changes on individuals, social groups and communities, SIA informs regulation, planning and management of a development project (Vanclay and Esteves 2024). Yet, despite the increasing emphasis on community engagement and social licence to operate (Prno and Slocombe 2012; Cesar and Jhony 2021), many SIAs struggle to meaningfully capture the values that local and Indigenous communities associate with pre-mining landscapes, especially those that are intangible, relational and culturally embedded. Because many of these values are not measurable or quantifiable, they often remain undocumented, misunderstood and underappreciated in the early stages of project planning, which limits their influence on development decisions and long-term closure visions.

As extractive industries move towards more inclusive and sustainable approaches, there is growing recognition of the need for these industry transitions to integrate diverse forms of knowledge (including local, experiential and place-based knowledge) into environmental and social planning. Amid these shifts, public participation geographic information systems (PPGIS) offer a promising, yet underutilized, method for surfacing and spatializing the social meanings and attachments people hold to their landscapes prior to mining. By enabling communities to map their values directly onto geographic space, PPGIS can help make invisible values visible, creating a shared, evidence-based foundation for dialogue, planning and future restoration thereby enriching SIA reports.

This perspective article explores the potential for PPGIS to become a vital tool within SIA, aligned with its core principles (Vanclay 2003). It offers value not only as a participatory engagement tool, but as a means of grounding mine development and closure planning in the lived experiences and priorities of local communities (Svobodova et al. 2025c). This article approaches SIA primarily as a regulatory, planning and management tool (e.g. Vanclay and Esteves 2024), rather than as a method of social research (e.g. Sairinen et al. 2021). We argue that that PPGIS can uniquely address the challenge of incorporating intangible, placed-based values into SIAs for mining projects. By enabling communities to spatially map what matters to them, PPGIS makes otherwise invisible meanings visible to decision-makers, thereby enriching SIA reports and supporting more inclusive and sustainable development and closure planning across the mine life cycle. This approach is a powerful tool to enhance public agency in the SIA process by integrating local knowledge into formal planning processes, moving beyond conventional, often one-way, information flows in SIAs.

## The problem: Intangible values in impact assessment

From its origins in the early 1970s, as a cursory inclusion in EIAs, SIA has become a forward-looking study that is part of the regulatory approval process in many countries (Esteves et al. 2012). SIA seeks a comprehensive understanding of the socio-economic risks and benefits of a development project so that the social consequences of infrastructure and resource developments are fully considered and well managed (Vanclay 2003; Esteves et al. 2012; Joyce et al. 2018). For decades, SIA has been the main policy instrument and planning tool for evaluating and managing the social aspects of various development projects (O’Faircheallaigh 2009; Vanclay et al. 2015; Joyce et al. 2018). SIAs for these purposes marry various social science disciplines, including sociology, political science, economics, demography and human geography with practice-oriented organizational and community analysis (Freudenberg 1986; Rickson et al. 1990). However, resulting reports are often fragmented rather than holistic (Hartz-Karp and Pope 2011) and landscape and spatial studies have proven less common (Ang et al. 2023). In parallel, there has been growing recognition that social and environmental assessments need to unite analytical with participatory methodologies since information on hard to elicit social values is best generated by community participation (Hartz-Karp and Pope 2011; Kenter 2016). Participatory processes provide SIA with “full and robust information on the populations that will be affected, on the nature of impacts, and on the likely efficacy of mitigation strategies” as well as first-hand information about the fears, aspirations and values of affected populations (O’Faircheallaigh 2024: 150).

Public participation is a key component of environmental decision making and impact assessment to collate knowledge and perspectives from diverse stakeholders (Vanclay 2003; Burdett 2024). Public stakeholders broadly include individuals, groups, businesses, professional organizations and government departments who are affected by, or interested in, the planning, operation and legacy of a proposed project (Lambropoulos 2010). Involving these members of the public in SIA responds to the common community aspiration to influence development projects that affect their economic, social and cultural wellbeing (O’Faircheallaigh 2024). Public participation in SIA is credited with strengthening baseline data, reducing information disparities, increasing understanding of change and capacities to respond to change (especially of vulnerable and disadvantaged people) and thereby avoiding and mitigating negative impacts while enhancing positive benefits across the life cycle of developments (Esteves et al 2012). Despite its evolution as an important component of mining approvals and regulatory frameworks, and the consensus that public participation should be integral to the process (Vanclay 2024), SIA often remains limited in its capacity to recognize and account for the full spectrum of values that communities hold towards their landscapes (O’Faircheallaigh 2009; Altman and Low 1992). Specifically, public participation in SIA is criticized as falling short of the ideal of constructing “the best possible understandings and agreements given what is reasonably knowable to the participants at the time” (Webler and Tuler 2002: 183). This is partly due to information flows being one-way, rather than two-way exchanges, and to the quality and quantity of predominant forms of data (Meissner and Everingham 2021). Because most public engagement in traditional SIA methodologies is initiated by project proponents who also provide the bulk of project information, SIA is criticized for prioritizing technical, quantitative data and even exaggerating projected socio-economic impacts to secure approval of projects (O’Faircheallaigh 2024). This frequently compromises baseline data and results in significant information gaps about the intangible, place-based dimensions of landscapes that are deeply embedded in community identity and memory (O’Faircheallaigh 2024). Some conclude that “new communicative instruments” (Buchecker et al. 2003: 29) are needed to allow non-judgemental exchange of ideas and provide people with “satisfying opportunities to influence the development of their landscape” (Buchecker et al. 2003: 31). Such instruments would also serve purposes beyond formal regulatory approvals since public participation in SIAs is recognized as crucial to gaining public trust and what has been called a “social license to operate” (Vanclay 2024; Burdett 2024; Moffat and Zhang 2014).

Pre-mining landscapes often carry significant emotional, historic and symbolic meanings for local residents and Indigenous peoples (Kemp et al. 2023; Svobodova et al. 2023; Cross 2015). The literature commonly frames these meanings as expressions of landscape values (e.g. Gómez-Sal et al. 2003; Penning-Rowsell and Lowenthal 2022). Landscape values encompass the significance individuals attribute to the environments where they live, work, visit or establish other personal connections (Brown et al. 2020a). Some of these values stem from tangible benefits, such as economic gains or recreational enjoyment, while others take on a more intangible and abstract nature, like spiritual and aesthetic appreciation. Notably, research suggests that it is these abstract values, rather than the more material ones, that most strongly influence people’s place attachment and their attitudes towards landscape change and development (Buchecker et al. 2003: 37).

Landscape values are a form of relationship values, connecting people to features such as roads, buildings and vegetation (Stenseke 2018; Brown et al. 2020b). This viewpoint enables the assignment of specific locations to these values. It underscores the connections between how people perceive the importance of a value and the inherent value within the physical features of the landscape. These values include, but are not limited to, aesthetic, cultural, economic, historic, recreation and wilderness values as shown in Table 1 (modified from Brown et al. 2020b).

**Table 1:** Landscape value typology with operational definitions modified from Brown et al. (2020b)

Landscape value	Definition
Aesthetic	Places that contain attractive scenery, including sights, smells and sounds
Economic	Places with economic benefits, e.g. providing timber, fisheries and minerals
Recreation	Places for outdoor recreation
Life-sustaining	Places that help to produce, preserve, clean, and renew air, soil and water
Educational	Places where people can learn about the environment through observation and study
Spiritual	Places with special sacred, religious and spiritual significance
Historic	Places representing natural and human history
Future	Places that allow future generations to know and experience the area as it is now
Subsistence	Places that provide necessary food and supplies to sustain people’s lives
Therapeutic	Places that make people feel better physically and mentally
Cultural	Places that provide opportunities to express and appreciate art, music, history, and other pieces of human culture
Wilderness	Places that are wild, uninhabited or relatively untouched by humans
Social	Places for social interactions
Special places	Places that are special and valuable for identity reasons

Landscape values are rarely documented in ways that make them visible or legible to decision-makers (De Groot et al. 2010; Shalsi et al. 2024). As a result, these values are frequently excluded from early planning processes, leaving communities feeling unheard or misrepresented (Barianaki et al. 2024). This absence is especially problematic given that the pre-disturbance state of the landscape often shapes local visions of what “restoration” or “closure” should look like in the long term. It can also result in missed opportunities to design post-mining landscapes that support cultural continuity and intergenerational knowledge transmission (Everingham et al. 2020).

Integrating these intangible and spatially explicit values into formal processes remains a methodological and institutional challenge (see e.g. Rawluk et al. 2019; Barianaki et al. 2024). Data on cultural ecosystem services (Cook et al. 2019; Svobodova et al. 2025a), place attachment (Svobodova et al. 2021) and other emotional ties to land are often perceived as “soft”, anecdotal or incompatible with technical planning tools. This disconnect is particularly evident in SIAs, which focus exclusively on value in financial terms thereby, as Measham and colleagues (2022: 5) warn, “ignoring a wider range of considerations which speak to things that matter to people (i.e. “values”)”. Furthermore, traditional consultation methods, such as public meetings or surveys, may not elicit the spatial specificity needed to inform land use decisions, design of mine footprints or closure plans. Kenter (2016) and Hartz-Karp and Pope (2011) see knowledge exchange, interrogating information, discussion of values and deliberation in groups as a fundamental part of forming reasoned and equitable landscape and development plans. Such analytical–deliberative approaches include participatory modelling and mapping and other non-monetary techniques using visualization such as colour maps, graphic illustrations and virtual tours as communication aids (Pettit et al. 2011; O’Faircheallaigh 2024). Recent technological advances enable 2-D and 3-D interaction with such visualizations (Pettit et al. 2011).

When these diverse values are missed or dismissed, the consequences can persist well beyond the operational phase of the mine. Communities may feel a sense of loss or disconnection from transformed environments, and legacy issues can emerge if closure strategies fail to resonate with local understandings of what matters in the landscape (for Indigenous communities, see Holcombe et al. 2025). In some cases, this can erode trust and social licence, resulting in conflict, prolonged negotiations and resistance to rehabilitation efforts (see e.g. Svobodova et al. 2025b).

This persistent gap calls for new tools and approaches that can help surface and integrate these intangible values early in the mine life cycle and support a more holistic, participatory approach to SIA and overall closure planning.

## The potential of PPGIS in SIA

PPGIS is a participatory mapping method that combines spatial data with local knowledge to capture how communities value and experience specific places (Brown and Weber 2013). The term “public participation GIS” was conceived in 1996 at the meeting of the National Center for Geographic Information and Analysis in the USA to describe how GIS technology could support public participation for a variety of applications with the goal of greater inclusion and empowerment of the public in decision-making processes (Brown and Pullar 2012). The approach typically involves engaging stakeholders through surveys, workshops or online platforms where participants mark on digital or paper maps locations that hold social, cultural, recreational and spiritual significance. These mapped inputs are then analysed alongside other spatial and demographic data to reveal patterns of use, attachment and meaning. By translating intangible values into spatially explicit information, PPGIS provides decision-makers with a more nuanced understanding of local contexts (Pettit et al. 2011; Korpilo et al. 2022; Ang et al. 2023).

PPGIS has been increasingly applied in urban and rural studies to understand the qualities of living environments, support housing development planning and monitor responses to environmental change. For example, it has been used to map place attachment in urban redevelopment (Wang 2021), identify green space values in metropolitan planning (Ives et al. 2017) and assess ecosystem services (Brown and Fagerholm 2015). In nature conservation, PPGIS has helped identify ecologically significant areas for biodiversity management (Brown and Weber 2013). A PPGIS-based prioritization model has been developed to identify high-priority areas requiring urgent planning attention for future developments (Kyttä et al. 2023). It has also been applied to reduce flood vulnerability in areas affected by cyclones by identifying locally known risk zones (Cruz Bello et al. 2018).

In the context of SIA, PPGIS hold untapped potential. In jurisdictions such as Australia, Canada and the European Union, regulatory frameworks require project proponents to assess and mitigate social impacts throughout the life of major projects, including mining developments. For example, in the Australian state of Queensland, SIAs are a statutory requirement for resource projects located within 125 kms of a regional community (Queensland Government 2018). Similarly, international standards such as those issued by the International Council on Mining and Metals (ICMM 2025) emphasize the importance of engaging with affected communities, respecting cultural values and identifying vulnerable groups. Despite these regulatory requirements, SIAs often fall short in capturing spatially nuanced or qualitative values of pre-disturbance landscapes. This is especially problematic in mining contexts, where landscapes undergo radical transformation and where the loss of culturally significant areas can have lasting social consequences (Wensing 2021; Svobodova et al. 2021, 2023). PPGIS offer a way to fill this gap. By integrating spatial methods with participatory processes, it enables early identification of culturally and socially important sites. Whether these are locations of daily use, symbolic meaning or collective memory. This can support more robust baseline studies and ensure that place-based values are considered in the design of mine project footprints, infrastructure siting and access arrangements. The importance of such approaches is underscored by high-profile failures, such as the destruction of ancient Indigenous rock shelters in the Juukan Gorge, Western Australia, by Rio Tinto in May 2020. In that instance, prioritizing production imperatives and regulatory compliance over Indigenous identity, spiritual connections and cultural values led to irreversible heritage loss (Wensing 2021; Kemp et al 2023).

From a compliance perspective, incorporating PPGIS into SIA demonstrates proactive and good-faith engagement with community stakeholders. It can serve as tangible evidence that community values were documented, considered and integrated into decision-making. This is a factor that can be critical for project approvals and is now recognized as crucial to gaining public trust (Svobodova et al. 2025c; Moffat and Zhang 2014). Furthermore, PPGIS bridge the methodological and regulatory gap between qualitative and quantitative data by translating local values and narratives into spatially explicit GIS datasets. These datasets can then be integrated into formal impact assessments and used to co-design closure scenarios that better respect the social fabric of affected regions.

## Embedding PPGIS across the mine life cycle

**Fig. 1:**
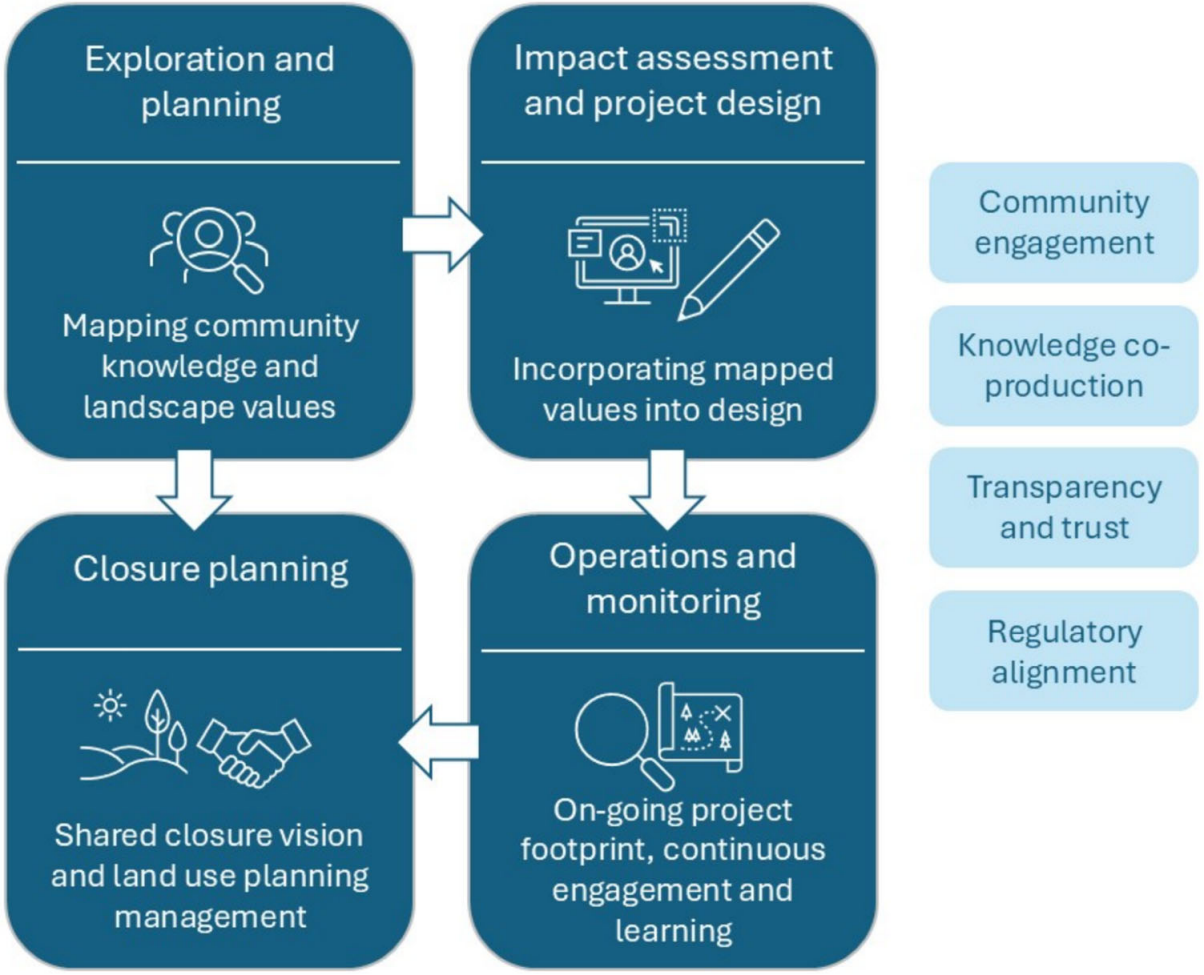
Diagram of mine life cycle with PPGIS implementation

To fully realize the potential of PPGIS in mining contexts, it should be integrated across the entire mine life cycle (Fig. 1), not as a one-off engagement tool, but as a continuous, adaptive process. PPGIS use must be aligned with regulatory milestones, but more importantly, it must be grounded in an ethos of co-producing knowledge. This means treating communities not as passive consultees but as active partners in shaping the social and spatial trajectory of mine development and closure. Approaches should include iterative mapping sessions, inclusive facilitation and transparent communication of how community input influences decisions at each stage.

### Exploration and planning

The application of PPGIS during exploration or pre-feasibility phases allows communities to spatially express values, uses and attachments before key decisions about project design and location are made. Mapping local knowledge and place-based meanings at this stage helps proponents avoid or minimize impacts on culturally sensitive areas. It also supports the identification of shared benefits, such as protecting heritage landscapes or aligning infrastructure planning with community priorities.

### Impact assessment and project design

As part of SIA, PPGIS can deepen the understanding of potential spatial impacts and social risks. It enables participatory identification of areas of concern, facilitates scenario-based discussions and supports the co-design of mitigation strategies. Incorporating participatory maps into design choices enhances transparency and shows respect for community perspectives, often improving trust and legitimacy.

### Operations and monitoring

Throughout the operational phase, PPGIS can be revisited periodically to track changes in how communities use, value and relate to the modified landscape and its progressive rehabilitation. By comparing maps over time, companies can identify shifting perceptions or emerging concerns and respond with adaptive management strategies. This iterative use of PPGIS supports proactive engagement, continuous learning and the ability to address unforeseen issues as they arise.

### Closure planning and post-mining transition

In the closure phase, early-stage PPGIS outputs can serve as a valuable benchmark to assess whether rehabilitation plans align with initial community values and aspirations. When integrated into closure visioning and post-mining land use planning, participatory maps offer a way to ensure that restored landscapes reflect social significance. This approach helps maintain continuity in local identity and supports intergenerational knowledge about place.

## Practical considerations and ethical challenges

While the promise of PPGIS is strong, with potential for a radical and transformative approach to SIA from a community, rather than developer perspective, its implementation comes with both practical and ethical considerations.

From a methodological standpoint, challenges arise around scale, representativeness, data quality, and technical accessibility. Not all participants may have equal familiarity with GIS software or spatial reasoning. This raises questions such as: Who participates in mapping? What tools are used? How are maps interpreted, validated and weighed against other inputs? The choice of method, whether digital mapping platforms, paper-based sketch mapping, mobile apps, or facilitated group sessions, should be adapted to participants’ technological comfort levels and the local context. This applies especially to more remote and sparsely populated areas, where mineral deposits are often located and where services may be limited. There is a lack of empirical research evaluating the effectiveness of PPGIS from a communications and engagement perspective, particularly in relation to its influence on decision-making processes and tangible project outcomes (Sieber 2006; Pettit et al. 2011; Brown and Kyttä 2018). Pettit et al. (2011) stressed the need for an effective community engagement plan in using PPGIS for collaborative exploration of future landscape scenarios. Additionally, Brown and Kyttä (2018) emphasize the need for a clear purpose, building trust, making informed decisions and adapting to complex, diverse contexts with appropriate indicators.

To address skill disparities, capacity-building may be required. This may include training sessions or facilitated workshops where community members can co-create spatial data with guidance. A mixed-methods approach that blends PPGIS with qualitative techniques such as interviews, focus groups, photovoice and ethnographic insight can help give voice to those less comfortable with maps.

There are also ethical and cultural sensitivities in mapping values, especially in contexts involving Indigenous peoples, customary land tenure and sacred sites. Spatializing such knowledge may be seen as intrusive or inappropriate, and concerns around data privacy, consent and ownership must be handled with great care. PPGIS practitioners must ensure informed consent, follow community protocols for knowledge sharing, and be transparent about where and how spatial data will be stored and used. PPGIS initiatives may require skilled facilitation since they do not per se overcome challenges of equitably encompassing the full diversity of experiences and values, especially where dispersed participants are unfamiliar with common consultation methods let alone sophisticated technological ones.

Finally, there is the issue of influence and accountability. Participatory mapping raises expectations that expressed values will shape outcomes. If community-generated maps are ignored or deprioritized in decision-making, it can deepen mistrust and cause harm. For PPGIS to maintain credibility, there must be clear communication about how input will inform decisions, along with visible follow-through in planning, design and closure processes.

## Conclusion

As the mining sector grapples with increasing pressure to demonstrate social legitimacy and inclusive governance, the ability to recognize and respond to the full range of landscape values becomes essential. PPGIS offer a method to visualize, document and incorporate these values in ways that are spatially explicit, culturally respectful and operationally relevant.

For SIA practitioners, regulatory bodies and mining companies, the integration of PPGIS can improve the quality and legitimacy of impact assessments, while also supporting longer-term goals of social cohesion, place attachment and meaningful rehabilitation. It aligns with international good practice, complements existing regulatory frameworks, and reflects a broader shift towards participatory, just and value-driven planning (Korpilo et al. 2022). We argue that PPGIS can provide a richer, more holistic approach to assessing social impacts, one consistent with key principles of SIA and harmonizing technocratic and participatory methods as advocated by Hartz-Karp and Pope (2011). By utilizing technical information (in this case maps and details of the concrete environment) as an input to the participatory process rather than as an output of the SIA and harnessing local knowledge, PPGIS provides a promising techniques for achieving informed and socially acceptable landscape planning. This improves on the typical SIA anticipating social impacts for regulatory approval because “Rather than an engagement ‘event’ there needs to be a range of initiatives that enable the technical knowledge of experts and the ‘practical wisdom’ of everyday people to collectively frame the issues, gather the information, and oversee the process to ensure it is comprehensive and fair” (Hartz-Karp and Pope 2011: 26).

Decades ago, Buchecker et al (2003:43) observed that “Participation in shaping the world is a nostalgic as well as an utopian concept, but the new, communicative form of it has not yet been found”. PPGIS may fulfil the dream as more than a technical tool, but also a medium for dialogue. It connects communities, planners and regulators around shared understandings of place. In the context of mine development and closure, this means planning not just for landscapes, but with the people who live in and care for them.
